# Comparison of the clinical effect of empagliflozin on glycemic and non-glycemic parameters in Japanese patients with type 2 diabetes and cardiovascular disease treated with or without baseline metformin

**DOI:** 10.1186/s12933-021-01352-0

**Published:** 2021-07-31

**Authors:** Atsushi Tanaka, Michio Shimabukuro, Hiroki Teragawa, Yosuke Okada, Toshinari Takamura, Isao Taguchi, Shigeru Toyoda, Hirofumi Tomiyama, Shinichiro Ueda, Yukihito Higashi, Koichi Node, the EMBLEM Investigators

**Affiliations:** 1 Department of Cardiovascular Medicine, Saga University, 5-1-1 Nabeshima, Saga, 849-8501 Japan; 2 Department of Diabetes, Endocrinology, and Metabolism, Fukushima Medical University, Fukushima, Japan; 3 Department of Cardiovascular Medicine, JR Hiroshima Hospital, Hiroshima, Japan; 4 First Department of Internal Medicine, School of Medicine, University of Occupational and Environmental Health, Kitakyusyu, Japan; 5 Department of Endocrinology and Metabolism, Kanazawa University Graduate School of Medical Sciences, Kanazawa, Japan; 6 Department of Cardiology, Dokkyo Medical University Saitama Medical Center, Koshigaya, Japan; 7 Department of Cardiovascular Medicine, Dokkyo Medical University School of Medicine, Mibu, Japan; 8 Department of Cardiology, Tokyo Medical University, Tokyo, Japan; 9 Department of Clinical Pharmacology and Therapeutics, University of the Ryukyus, Nishihara, Japan; 10 Department of Cardiovascular Regeneration and Medicine, Research Institute for Radiation Biology and Medicine, Hiroshima University, Hiroshima, Japan

**Keywords:** Type 2 diabetes, Cardiovascular disease, Sodium-glucose cotransporter 2 inhibitor, Metformin

## Abstract

**Background:**

The most recent treatment guidelines for type 2 diabetes (T2D) recommend sodium-glucose cotransporter 2 (SGLT2) inhibitors should be considered preferentially in patients with T2D with either a high cardiovascular risk or with cardiovascular disease (CVD), regardless of their diabetes status and prior use of conventional metformin therapy. Whether the therapeutic impact of SGLT2 inhibitors on clinical parameters differs according to the use of metformin therapy however remains unclear.

**Methods:**

The study was a post hoc analysis of the EMBLEM trial (UMIN000024502). All participants (n = 105; women 31.4%; mean age 64.8 years) had both T2D and CVD and were randomized to either 24 weeks of empagliflozin 10 mg daily or placebo. Analysis of the data assessed the effect of empagliflozin on changes from baseline to 24 weeks in glycemic and non-glycemic clinical parameters, according to the baseline use of metformin.

**Results:**

Overall, 53 (50.5%) patients received baseline metformin. In the 52 patients treated with empagliflozin (48.1% with baseline metformin), the decrease in systolic blood pressure from baseline levels was greater in patients receiving metformin, compared to that observed in metformin-naïve patients (group difference − 8.5 [95% confidence interval (CI) − 17.7 to 0.6 mmHg], *p* = 0.066). Reduction in body mass index (BMI) was significantly greater in patients receiving baseline metformin, relative to nonusers (− 0.54 [95% CI − 1.07 to − 0.01] kg/m^2^, *p* = 0.047). The group ratio (baseline metformin users vs. nonusers) of proportional changes in the geometric mean of high-sensitivity Troponin-I (hs-TnI) was 0.74 (95% CI 0.59 to 0.92, *p* = 0.009). No obvious differences were observed in glycemic parameters (fasting plasma glucose, glycohemoglobin, and glycoalbumin) between the baseline metformin users and nonusers.

**Conclusion:**

Our findings suggest 24 weeks of empagliflozin treatment was associated with an improvement in glycemic control, irrespective of the baseline use of metformin therapy. The effects of empagliflozin on reductions in BMI and hs-TnI were more apparent in patients who received baseline metformin therapy, compared to that observed in metformin-naïve patients.

*Trial registration* University Medical Information Network Clinical Trial Registry, number 000024502

## Introduction

Based on accumulated clinical evidence and demonstration of safety and efficacy, metformin has become recognized as an established glucose-lowering agent for the initial treatment of type 2 diabetes (T2D) [1]. In the latest European Society of Cardiology (ESC) Guidelines on diabetes, pre-diabetes, and cardiovascular diseases developed in collaboration with the European Association for the Study of Diabetes (EASD), it was also recommended that metformin should be considered as first-line therapy in patients with T2D, especially in overweight patients without cardiovascular disease (CVD) and those with a moderate cardiovascular risk [2]. Recent cardiovascular outcome trials (CVOTs) on several classes of glucose-lowering agents, including sodium-glucose cotransporter 2 (SGLT2) inhibitors and glucagon-like peptide-1 receptor agonists, have shown that these agents reduced cardiovascular events and death in patients with T2D at high cardiovascular risk or with established CVD. This led to a recent groundbreaking revision of treatment guidelines to use either agents in these patients, independent of baseline use of metformin [1, 2]. Given the previous position of metformin in diabetes care, this was likely a marked paradigm shift in the selection of glucose-lowering agents.

In the CVOTs completed before the development of such a paradigm shift, almost all participants (i.e., more than two-thirds) received baseline metformin therapy in accordance with conventional treatment strategy for diabetes care and had the agents investigated in this study added to metformin therapy [3–12]. This raised the clinical question as to whether or not the cardiovascular benefits of the agents would be expected even in patients with T2D at high cardiovascular risk or those with CVD, independent of the effects of baseline metformin. However, subsequent analyses demonstrated that treatment with these agents consistently improved cardiovascular outcomes regardless of the baseline use of metformin [13–17]. These findings also support recent updated treatment guidelines for better outcomes especially in patients with T2D either at high cardiovascular risk or with CVD. However, only limited clinical data are currently available regarding differences in the effect of these agents on glycemic and non-glycemic parameters in this patient population. Identifying these differences may help to achieve optimal selection of glucose-lowering agents for secondary prevention of CVD in actual clinical settings.

The EMBLEM (Effect of Empagliflozin on Endothelial Function in Cardiovascular High Risk Diabetes Mellitus: Multi-Center Placebo-Controlled Double-Blind Randomized) trial had the primary aim of determining whether 24 weeks of empagliflozin treatment affected peripheral endothelial function in patients with T2D and established CVD [18, 19]. In that trial, about one-half of the participants did not receive metformin therapy at baseline. Therefore, the current post hoc analysis of the EMBLEM trial examined whether the effect of 24 weeks of empagliflozin treatment on glycemic and non-glycemic clinical parameters in patients with T2D and CVD differed according to the use of baseline metformin treatment.

## Methods

### Study design

The EMBLEM trial (UMIN000024502) was an investigator-initiated, multi-center, placebo-controlled, double-blinded, randomized-controlled trial undertaken in 16 centers in Japan. The details of the study design and primary results have been reported previously [18–20]. Briefly, eligible patients were ≥ 20 years old, with T2D, and a glycohemoglobin (HbA1c) level ranging from 6.0 and 10.0%, who were clinically stable without changes in T2D therapy for at least one month before consent, and had a previous history of at least one established CVD (coronary artery disease, stroke, peripheral artery disease, presence of known coronary artery stenosis (≥ 50%), or heart failure (HF) with a New York Heart Association classification I to III and clinically stable by the use of HF-medications for at least one month before consent). Key exclusion criteria were type 1 diabetes, a history of diabetic ketoacidosis or diabetic coma within the last 6 months, severe renal dysfunction (estimated glomerular filtration rate (eGFR) < 45 mL/min/1.73 m^2^ or undergoing dialysis), serious liver dysfunction, a history of atherosclerotic CVD within 3 months before consent, and prior use of a SGLT2 inhibitor within one month before consent.

The participants were assigned randomly to either 10 mg of daily empagliflozin or to placebo, using a web-based minimization system that balances for HbA1c (< 7.0 or ≥ 7.0%), age (< 65 or ≥ 65 years), systolic blood pressure (BP) (< 140 or ≥ 140 mmHg), and current smoking status at the time of screening. The participants underwent scheduled visits after 4, 12, and 24 weeks for dispensing of drugs and assessment of study endpoints. Although no specific goal of glycemic control was set in the EMBLEM trial, all participants were to be treated in accordance with the local treatment guidelines for T2D of the Japan Diabetes Society at that time. Each participant’s background medications, including glucose-lowering therapy, were in principle unchanged during the trial. However, if the therapeutic effect of the medications was insufficient, the addition of glucose-lowering agents other than SGLT2 inhibitors or an increased dosage of background medications were allowed at the judgement of the local investigator.

The ethical committees of the participating institutions approved the study protocol. Written, informed consent for participation in the study was obtained from all the subjects. This study was performed in accordance with the Helsinki Declaration of 1964 and its later amendments.

### Outcome measures

The details of the original outcome measures in the EMBLEM trial have been reported previously [20]. The main endpoints in the post hoc analysis using data obtained from the trial were changes from baseline to week 24 in glycemic parameters (fasting plasma glucose [FPG], HbA1c, and glycoalbumin [GA]) and non-glycemic parameters, including systolic and diastolic BP, heart rate (HR), double product (systolic BP × HR), body mass index (BMI), N-terminal pro-brain natriuretic peptide (NT-proBNP), and high-sensitivity Troponin-I (hs-TnI). Of the laboratory markers, the assays of GA (SRL, Inc., Tokyo, Japan), NT-proBNP (SRL, Inc., Tokyo, Japan), and hs-TnI (Abbott Japan LLC, Tokyo, Japan) were performed at central laboratories. The post hoc analysis assessed the effect of empagliflozin on these variables according to the use or nonuse of baseline metformin.

### Statistical analysis

All the analyses were conducted on the full analysis set, which included all participants who had received at least one dose of the study medication after randomization and who did not have any serious violation of the protocol. Baseline demographics and characteristics were expressed as numbers (percentages) for categorical variables and as means ± standard deviation for continuous variables. Data on NT-proBNP and hs-TnI were expressed as geometric mean (95% confidence interval [CI]), and the proportional changes from baseline to week 24 calculated based on a natural logarithmic scale. Inter-group differences and ratios were compared using Welch’s t tests for continuous variables or Fisher’s exact test for categorical variables. All statistical analyses were performed using SAS software version 9.4 (SAS Institute, Cary, NC, USA). A two-sided significance level of *p* < 0.05 was used for all assessments, with no adjustment for multiplicity being used in the analyses.

## Results

### Baseline characteristics

A detailed participant flow-chart and the overall baseline characteristics of the EMBLEM trial have been reported elsewhere [18, 19]. Briefly, of the 117 patients randomized, 105 patients (64.8 ± 10.4 years old; women 33 [31.4%]; HbA1c 7.2 ± 0.8%; diabetes duration 13.3 ± 11.1 years) were included in the full analysis set (empagliflozin group n = 52, placebo group n = 53). Overall, 69 (65.7%), 18 (17.1%), and 79 (75.2%) patients were taking an angiotensin-converting enzyme or angiotensin receptor blocker, diuretic, and statin therapy, respectively. The majority of patients (69.5%) were taking a dipeptidyl peptidase-4 (DPP-4) inhibitor. Twenty-four patients (22.9%) were taking one type of glucose-lowering medication, while 67 patients (63.8%) were taking ≥ 2 types of these medications.

At baseline, a total of 53 (50.5%) patients were receiving metformin therapy, with 25 (48.1%) in the empagliflozin group and 28 (52.8%) in the placebo group. As shown in Table 1, the baseline characteristics were almost similar between the randomization groups, irrespective of the use of metformin at baseline.

**Table 1:** Baseline characteristics stratified by the use of baseline metformin

Variables	Metformin			No metformin		
	Empagliflozin (n = 25)	Placebo (n = 28)	*P* value*	Empagliflozin (n = 27)	Placebo (n = 25)	*P* value*
Age (yr)	64.6 ± 11.1	62.1 ± 10.1	0.400	66.3 ± 11.1	66.3 ± 9.3	0.983
Women	9 (36.0)	11 (39.3)	1.000	7 (25.9)	6 (24.0)	1.000
Diabetes duration (yr)	14.8 ± 10.1	12.9 ± 7.9	0.478	12.2 ± 16.2	13.2 ± 9.1	0.810
eGFR (mL/min/1.73 m^2^)	65.9 ± 10.9	73.0 ± 14.0	0.044	67.9 ± 13.8	65.1 ± 12.9	0.437
eGFR < 60 mL/min/1.73 m^2^	7 (28.0)	4 (14.3)	0.313	8 (29.6)	10 (40.0)	0.562
History						
Hypertension	20 (80.0)	19 (67.9)	0.365	21 (77.8)	17 (68.0)	0.536
Dyslipidemia	19 (76.0)	20 (71.4)	0.763	20 (74.1)	18 (72.0)	1.000
Cerebrovascular disease	2 (8.0)	6 (21.4)	0.256	4 (14.8)	9 (36.0)	0.112
Cardiovascular disease	24 (96.0)	24 (85.7)	0.355	26 (96.3)	20 (80.0)	0.094
Heart failure	12 (48.0)	12 (42.9)	0.786	11 (40.7)	7 (28.0)	0.392
Medication						
ACE inhibitor or ARB	15 (60.0)	17 (60.7)	1.000	16 (59.3)	21 (84.0)	0.068
Beta-blocker	9 (36.0)	7 (25.0)	0.550	10 (37.0)	12 (48.0)	0.575
Diuretic	2 (8.0)	3 (10.7)	1.000	6 (22.2)	7 (28.0)	0.752
Statin	22 (88.0)	18 (64.3)	0.059	21 (77.8)	18 (72.0)	0.752
Insulin	3 (12.0)	2 (7.1)	0.658	2 (7.4)	3 (12.0)	0.662
Sulfonylurea	7 (28.0)	7 (25.0)	1.000	1 (3.7)	5 (20.0)	0.094
Alpha-glucosidase inhibitor	4 (16.0)	6 (21.4)	0.732	4 (14.8)	2 (8.0)	0.670
Thiazolidinedione	7 (28.0)	10 (35.7)	0.572	5 (18.5)	3 (12.0)	0.705
DPP-4 inhibitor	17 (68.0)	19 (67.9)	1.000	20 (74.1)	17 (68.0)	0.762

Data are expressed as n (%) or mean ± SD

*ACE* angiotensin-converting enzyme, *ARB* angiotensin receptor blocker, *DPP-4* dipeptidyl peptidase-4, *eGFR* estimated glomerular filtration rate

^*^ Empagliflozin vs. placebo

### Changes in clinical parameters according to baseline metformin use

Changes from baseline to week 24 in clinical parameters according to the baseline use of metformin and randomization group are shown in Table 2. In patients receiving baseline metformin, the magnitude of reduction in the levels of systolic BP, double product, BMI, HbA1c, and GA were greater in patients treated with 24 weeks of empagliflozin, compared to those on placebo. In contrast, examination of the magnitude of changes in clinical parameters in metformin-naïve patients at baseline showed these were similar in the randomization groups, except for FPG. Irrespective of the use of baseline metformin, no significant randomization-based group ratios of proportional changes in NT-proBNP (metformin users, 1.11 [95% CI 0.78 to 1.58], *p* = 0.551; nonusers, 1.11 [95% CI 0.76 to 1.64], *p* = 0.579) and hs-TnI (metformin users, 0.93 [95% CI 0.74 to 1.16], *p* = 0.490; nonusers, 0.96 [95% CI 0.73 to 1.27], *p* = 0.788) were observed between the randomization groups.

**Table 2:** Changes from baseline to week 24 in clinical parameters, grouped according to baseline metformin use

Variables	Metformin			No metformin		
	Empagliflozin (n = 25)	Placebo (n = 28)	*P* value*	Empagliflozin (n = 27)	Placebo (n = 25)	*P* value*
Systolic blood pressure (mmHg)						
Baseline	136.0 ± 15.9	134.5 ± 15.9	0.733	129.9 ± 14.1	131.4 ± 12.9	0.690
Week 24	123.8 ± 14.4	132.1 ± 12.5	0.031	126.0 ± 14.6	128.8 ± 14.6	0.503
Change from baseline to week 24	− 12.0 ± 14.9	− 2.4 ± 10.9	0.012	− 3.5 ± 17.1	− 1.9 ± 13.7	0.718
Diastolic blood pressure (mmHg)						
Baseline	77.3 ± 12.3	75.5 ± 8.6	0.557	75.6 ± 11.0	74.3 ± 10.6	0.671
Week 24	72.0 ± 10.8	74.6 ± 11.7	0.420	73.2 ± 7.6	74.9 ± 11.0	0.536
Change from baseline to week 24	− 5.0 ± 8.2	− 1.0 ± 9.5	0.110	− 2.5 ± 9.1	0.8 ± 10.5	0.243
Heart rate (bpm)						
Baseline	75.3 ± 12.7	71.2 ± 10.1	0.202	72.5 ± 14.0	72.7 ± 9.7	0.952
Week 24	72.4 ± 11.6	70.4 ± 9.7	0.495	75.8 ± 19.5	71.6 ± 11.0	0.351
Change from baseline to week 24	− 2.3 ± 9.8	− 0.8 ± 7.2	0.547	3.1 ± 19.1	− 0.4 ± 9.8	0.410
Double product						
Baseline	10,166.64 ± 1683.93	9607.00 ± 1922.19	0.264	9499.78 ± 2440.30	9594.64 ± 1813.02	0.874
Week 24	8916.54 ± 1517.31	9344.29 ± 1781.22	0.354	9502.46 ± 2314.20	9214.26 ± 1811.87	0.628
Change from baseline to week 24	− 1147.13 ± 1693.95	− 262.71 ± 1082.76	0.034	3.46 ± 2711.37	− 223.91 ± 1400.61	0.710
Body mass index (kg/m^2^)						
Baseline	26.24 ± 6.11	27.41 ± 6.35	0.496	26.29 ± 4.04	26.11 ± 4.43	0.876
Week 24	25.40 ± 5.75	27.36 ± 6.03	0.236	25.94 ± 4.14	25.95 ± 4.69	0.995
Change from baseline to week 24	− 1.02 ± 0.83	− 0.05 ± 0.98	< .001	− 0.48 ± 1.03	− 0.17 ± 0.86	0.246
Fasting plasma glucose (mg/dL)						
Baseline	141.17 ± 21.40	150.46 ± 28.67	0.188	141.69 ± 28.26	141.75 ± 40.94	0.995
Week 24	121.17 ± 22.74	145.50 ± 41.50	0.011	134.70 ± 26.38	145.57 ± 44.97	0.324
Change from baseline to week 24	− 22.30 ± 19.86	− 4.96 ± 40.70	0.054	− 13.57 ± 23.50	4.26 ± 33.69	0.044
Glycohemoglobin (%)						
Baseline	7.30 ± 0.72	7.33 ± 0.65	0.864	7.02 ± 0.89	7.05 ± 1.08	0.916
Week 24	6.95 ± 0.61	7.52 ± 0.81	0.006	6.87 ± 0.67	6.95 ± 0.83	0.704
Change from baseline to week 24	− 0.35 ± 0.42	0.19 ± 0.62	< .001	− 0.15 ± 0.54	− 0.08 ± 0.79	0.703
Glycoalbumin (%)						
Baseline	18.50 ± 2.68	18.50 ± 2.83	0.996	17.78 ± 3.41	18.45 ± 3.97	0.541
Week 24	17.00 ± 2.40	18.73 ± 3.07	0.027	16.58 ± 3.26	17.97 ± 3.81	0.188
Change from baseline to week 24	− 1.51 ± 1.60	0.23 ± 1.68	< .001	− 1.23 ± 1.99	0.03 ± 2.67	0.087
NT-proBNP (pg/mL)						
Baseline	54.64 (32.35 to 92.28)	42.75 (27.33 to 66.88)	0.467	92.07 (52.02 to 162.96)	158.56 (79.24 to 317.25)	0.217
Week 24	51.23 (31.79 to 82.55)	37.24 (23.54 to 58.92)	0.326	85.19 (49.55 to 146.46)	138.01 (75.76 to 251.39)	0.223
Proportional change from baseline to week 24	0.97 (0.76 to 1.23)	0.87 (0.67 to 1.14)	0.551	1.03 (0.77 to 1.36)	0.92 (0.69 to 1.22)	0.579
hs-TnI (pg/mL)						
Baseline	3.64 (2.73 to 4.86)	3.20 (2.45 to 4.18)	0.501	5.02 (2.89 to 8.72)	8.78 (4.68 to 16.44)	0.174
Week 24	3.22 (2.33 to 4.45)	3.06 (2.26 to 4.14)	0.813	6.36 (3.30 to 12.26)	9.54 (5.07 to 17.95)	0.362
Proportional change from baseline to week 24	0.85 (0.71 to 1.03)	0.92 (0.82 to 1.04)	0.490	1.16 (1.02 to 1.32)	1.20 (0.94 to 1.54)	0.788

Data are expressed as mean ± SD or geometric mean (95% confidence interval) for NT-proBNP and hs-TnI

*hs-TnI* high-sensitivity Troponin-I, *NT-proBNP* N-terminal pro-brain natriuretic peptide

^*^ Empagliflozin vs. placebo

### Effect of empagliflozin in metformin users and nonusers

**Fig. 1:**
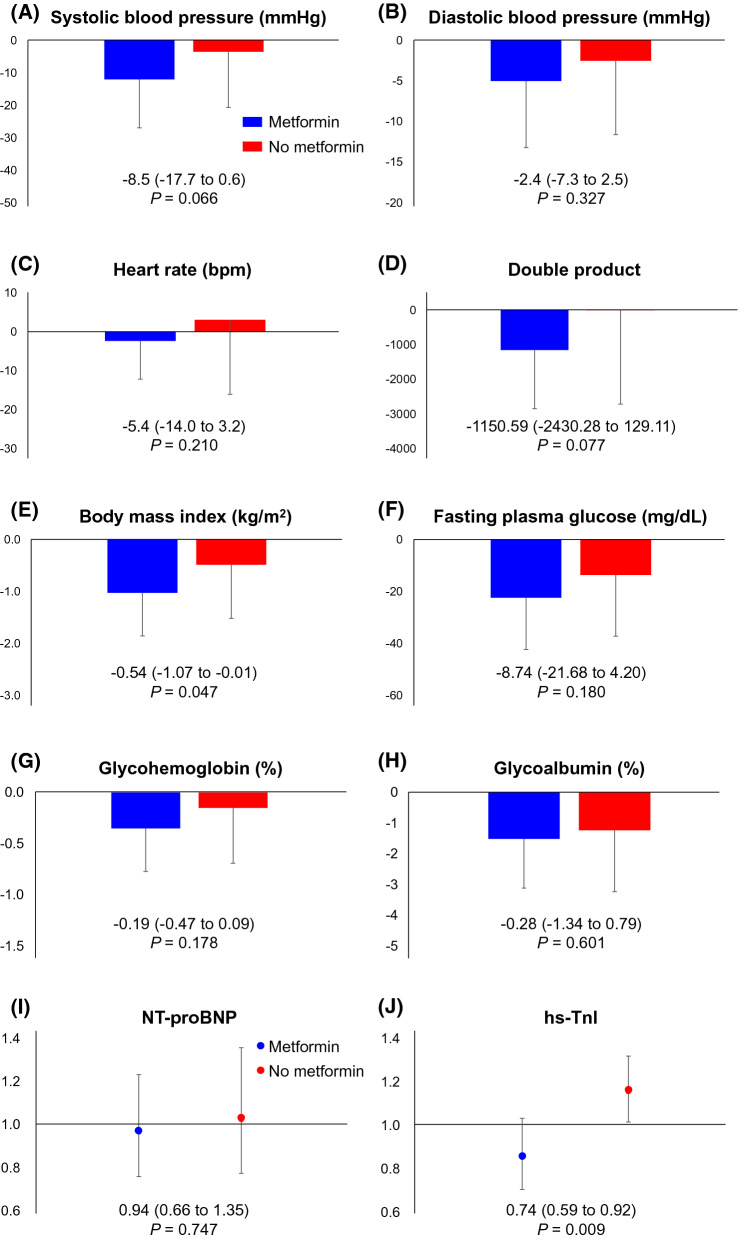
Change in clinical parameters from baseline to week 24 in patients treated with empagliflozin according to baseline use of metformin. Blue (red) indicates the group with (without) baseline metformin therapy. Values located at the bottom of each panel indicate the mean group difference (95% confidence interval) in the magnitude of change from baseline to week 24 in systolic blood pressure (**A**), diastolic blood pressure (**B**), heart rate (**C**), double product (systolic blood pressure × heart rate: **D** body mass index (**E**), fasting plasma glucose (**F**), glycohemoglobin (**G**), and glycoalbumin (**H**) or the mean group ratio (95% confidence interval) of the change ratio from baseline to 24 weeks in the geometric means of NT-proBNP (**I**) and hs-TnI (**J**). hs-TnI, high-sensitivity Troponin-I; NT-proBNP, N-terminal pro-brain natriuretic peptide

In the group treated with empagliflozin, the changes in systolic BP and double product from baseline to week 24 were greater in patients receiving baseline metformin therapy than those in metformin-naïve patients. However, these differences were not statistically significant (Fig. 1A, D). The reductions in BMI were significantly greater in patients who received baseline metformin therapy, compared to those who did not (Fig. 1E). No significant differences were observed between baseline metformin users and nonusers for the effect of empagliflozin treatment on the other parameters; HR, diastolic BP, and glycemic parameters (FPG, HbA1c, and GA) (Fig. 1B, C, F–H). The effect of empagliflozin on NT-proBNP concentration was also similar between the baseline metformin users and nonusers (Fig. 1I), while the group ratio (baseline metformin users vs. nonusers) of proportional changes in the geometric mean of hs-TnI was 0.74 (95% CI 0.59 to 0.92, *p* = 0.009: Fig. 1J).

## Discussion

This post hoc analysis of the EMBLEM trial on patients with T2D and established CVD, showed that the effects of 24 weeks of empagliflozin treatment on non-glycemic parameters, such as BMI and hs-TnI level, were more apparent in patients who had received baseline metformin therapy compared to metformin-naïve patients. In contrast, the impact of empagliflozin treatment on standard glycemic parameters was similar in patients who had received baseline metformin therapy to those who had not. To our knowledge, this is the first study in patients with T2D and CVD that assessed whether the effects of empagliflozin on clinical parameters differ according to the use of baseline metformin. Given the recent recommendation for SGLT2 inhibitors to be considered as key drugs for improving outcomes in patients with T2D at high cardiovascular risk or with CVD, regardless of previous metformin use [1, 2], the findings of our study may provide clinicians with novel insights on the effects of empagliflozin on a range of clinical parameters used frequently in the daily care of diabetes and cardiovascular disease, according to the patient’s prior use of metformin.

In the landmark trial of the United Kingdom Prospective Diabetes Study (UKPDS), metformin, relative to diet alone or other glucose-lowering agents such as sulfonylureas and insulin, was shown to reduce the risk of cardiovascular events and mortality in obese patients with newly diagnosed T2D [21]. The study also demonstrated that this early treatment effect was maintained, as a legacy effect, for as long as 10 years after the study intervention [22]. Based on its high degree of safety and efficacy and low-cost, metformin has been recommended over two decades as the first-line glucose-lowering agent for treating T2D. However, this recommendation was made before the use of modern and established cardiovascular protective drugs, such as statins and renin-angiotensin aldosterone system inhibitors. Accordingly, the majority of participants in earlier and recent CVOTs on newer glucose-lowering agents, including SGLT2 inhibitors, were administered metformin at baseline. Nonetheless, previous meta-analyses have reported heterogeneous and controversial effects of metformin on CVD and mortality, due mainly to a lack of good evidence obtained using current standards of care and trial methodology [23–25]. In addition, several important results from these CVOTs have led to the most recent guidelines recommending SGLT2 inhibitors as the preferred pharmacological therapy for specific patients, such as those with T2D and a high cardiovascular risk or with CVD, including HF and chronic kidney disease, regardless of their prior use of metformin [1, 2].

To date, several secondary observations and meta-analyses using data obtained from CVOTs on newer glucose-lowering agents have been conducted to assess the influence of baseline metformin therapy on the effect of these agents on cardiovascular outcomes [13–17]. A secondary analysis of the EMPA-REG OUTCOME (Empagliflozin Cardiovascular Outcome Event Trial in Type 2 Diabetes Mellitus Patients-Removing Excess Glucose) trial showed that the cardiovascular benefits of empagliflozin treatment in patients with T2D and CVD were unaffected by the use of baseline glucose-lowering agents, including metformin [15]. A meta-analysis on SGLT2 inhibitors also showed a consistent reduction in the risk of cardiovascular, renal, and mortality outcomes, irrespective of baseline use of metformin [16]. The finding that SGLT2 inhibitors are associated with a reduced risk of cardiovascular death or hospitalization for HF even in metformin-naïve patients with T2D at high cardiovascular risk or with CVD [17] supports the updated guidelines that recommend SGLT2 inhibitors as first-line therapy in this patient population [1, 2]. However, in such clinical settings, it remains uncertain whether SGLT2 inhibitors affect clinical parameters similarly or differently according to the use of background metformin therapy.

In the EMBLEM trial, one-half of the participants were metformin-naïve at baseline, with similar proportions in both randomization groups, while 70% of participants had been taking DPP-4 inhibitors at baseline. In Japan, the recommended approach when choosing between glucose-lowering agents is to consider the pathophysiology of the individual patient’s diabetes and therefore metformin therapy may not necessarily be the first-line pharmacological treatment for patients with T2D [26–28]. In such a situation, the current study on relatively well controlled patients with T2D showed that the addition of empagliflozin treatment was associated with similar improvements in glycemic parameters in patients with or without baseline metformin therapy. Our findings also partially support the possibility that SGLT2 inhibitors have an additive glycemic effect to that of DPP-4 inhibitors, regardless of whether the patient has received baseline metformin therapy [29, 30]. Therefore, the addition of empagliflozin to baseline glucose–lowering therapy, even in metformin users, may lead to improved glycemic control.

On the other hand, when considering recent positive CVOTs on SGLT2 inhibitors, the cardiovascular benefits of this class of drug are likely independent of their glucose-lowering effect [31]. It is well known that SGLT2 inhibitors have clinically multifaceted non-glycemic actions beyond their glucose-lowering action, such as body weight (BW) loss, a BP-lowering action, and cardiorenal protection, [32–34]. In the original randomized population of the EMBLEM trial, 24 weeks of empagliflozin treatment, relative to placebo, reduced BMI significantly and tended to decrease systolic and diastolic BP [18]. However, only a small amount of data is currently available on the assessment of the non-glycemic effects of SGLT2 inhibitors according to prior use of metformin therapy. In the current study the reduction in systolic BP and resultant double product, and BMI induced by empagliflozin was greater in patients who had received baseline metformin therapy compared to that observed in metformin-naïve patients. Although the precise reason(s) for these different effects remains unclear, the higher levels of systolic BP and a lower prescription rate for diuretics at baseline in patients on metformin compared with non-users may have enhanced the results in the present study. The different effects of empagliflozin on some parameters that we observed may therefore be associated with variabilities in the relevant backgrounds of the participants.

There is evidence that the effect of chronic metformin treatment on BW and BMI is to cause a mild reduction in BW, also in Japanese patients with T2D [35], although the detailed mechanism of this effect of metformin on BW loss has yet to be fully understood. In addition, Apolzan et al. reported recently that chronic metformin treatment was also effective for maintaining a reduction in BW over time in subjects with a high-risk of developing diabetes [36]. These results might indicate that the baseline use of metformin helps to enhance and retain the immediate BW-reducing effect of SGLT2 inhibitors in the present study. Furthermore, combination therapy of a SGLT2 inhibitor and metformin might have efficiently promoted BW loss through intrinsic insulin saving [37, 38], selective fat mass reduction [39, 40], and mitigation of compensatory overeating induced by chronic administration of SGLT2 inhibitors [41, 42]. Collectively, this combination therapy may provide synergistic benefits in some non-glycemic parameters, and as a consequence deliver a comprehensive therapeutic approach for diabetes-related complications, beyond that provided by lowering of glucose levels.

Interestingly, our analysis of established markers of cardiac stress and damage showed that the baseline levels of NT-proBNP and hs-TnI in patients who had received baseline metformin appeared to be lower than those in nonusers, although this difference was not statistically significant. The prevalence of a previous history of HF was similar between patients with or without baseline metformin therapy. This finding that the use of baseline metformin therapy might be associated with lower levels of these cardiac markers may, in part, support the possibility that metformin has robust cardioprotective effects via multifaceted molecular mechanisms, such as AMP-activated protein kinase-dependent and -independent signaling pathways [43]. In fact, the latest guidelines still recommend metformin therapy should be continued or included in patients with T2D and HF [1, 2]. Furthermore, empagliflozin treatment was associated with significantly greater reductions in hs-TnI concentrations in patients with baseline metformin therapy, compared to that observed in metformin-naïve patients. This finding raises the possibility that the protective effect of this combination therapy on micro-myocardial damage may, at least in part, be enhanced by several cardiometabolic actions, including a modulation of impaired cardiac insulin signaling [44]. However, the clinical impact of SGLT2 inhibitors on those cardiac biomarkers remains controversial [45]. Further research is therefore warranted in order to better understand which markers are most suitable for clinical monitoring of the cardiovascular effects of SGLT2 inhibitors.

The present study had several limitations. First, it was a post hoc analysis that used data obtained from the EMBLEM trial that was designed primarily to assess the effect of empagliflozin treatment, relative to placebo, on peripheral endothelial function. Second, although about one-half of the participants in the study were metformin-naïve at baseline, the clinical reasons why these patients did not receive metformin are unknown. Hence, some confounding factors with metformin use, such as renal function and severity of HF may have partially affected the impact of empagliflozin treatment on our study endpoints in the patients with or without baseline metformin therapy. Third, the small number of patients in the subgroups may not have provided sufficient statistical power to detect true group differences in the clinical parameters examined in the study. Furthermore, adjustments of potential confounding factors at baseline and changes in the glucose-lowering agents during the trial were not carried out due to the small sample size and limited clinical information. Finally, because the EMBLEM trial included only Japanese patients with T2D and CVD, who were clinically stable and met the study inclusion and exclusion criteria, further research is needed to assess whether the present findings are applicable to other ethnicities and/or different patient populations.

## Conclusion

This study in patients with T2D and CVD demonstrated that 24 weeks of empagliflozin treatment was associated with an improvement in glycemic control, irrespective of baseline use of metformin therapy. The effects of empagliflozin on reductions in BMI and hs-TnI were more apparent in patients who received baseline metformin, compared to that observed in metformin-naïve patients. Based on the most recent guidelines for T2D, especially for patients with increased cardiorenal risk, the clinical opportunity to prescribe SGLT2 inhibitors will likely increase, and further research is therefore needed to investigate the effect of these drugs on clinical parameters, taking into account the background situation of conventional glucose-lowering agents, such as metformin.

## Data Availability

The datasets analyzed during the current study are available from the corresponding author on reasonable request (tanakaa2@cc.saga-u.ac.jp).

## Abbreviations

BMI: Body mass index
BP: Blood pressure
BW: Body weight
CI: Confidence interval
CVD: Cardiovascular disease
CVOT: Cardiovascular outcome trial
DPP-4: Dipeptidyl peptidase-4
EASD: European Association for the Study of Diabetes
ESC: European Society of Cardiology
FPG: Fasting plasma glucose
GA: Glycoalbumin
HbA1c: Glycohemoglobin
HF: Heart failure
HR: Heart rate
hs-TnI: High-sensitivity Troponin-I
NT-proBNP: N-terminal pro-brain natriuretic peptide
SGLT2: Sodium glucose co-transporter 2
